# Development and Optimization of Freeze-Dried Eye Drops Derived From Plasma Rich in Growth Factors Technology

**DOI:** 10.1167/tvst.9.7.35

**Published:** 2020-06-25

**Authors:** Eduardo Anitua, María de la Fuente, Ignacio Alcalde, Cristina Sanchez, Jesús Merayo-Lloves, Francisco Muruzabal

**Affiliations:** 1 Biotechnology Institute (BTI), Vitoria, Spain; 2 University Institute for Regenerative Medicine and Oral Implantology - UIRMI (UPV/EHU-Fundación Eduardo Anitua), Vitoria, Spain; 3 Instituto Oftalmológico Fernández-Vega, Fundación de Investigación Oftalmológica, Universidad de Oviedo, Oviedo, Spain

**Keywords:** plasma rich in growth factors, freeze-dried eye drops, platelet rich plasma, lyoprotectants, PRP, wound healing

## Abstract

**Purpose:**

To investigate whether plasma rich in growth factors (PRGF) eye drops maintain their biological potential after a freeze drying process. The addition of a lyoprotectant like trehalose was also evaluated.

**Methods:**

Blood from three healthy donors was collected to obtain eye drops by PRGF technology. The resultant eye drops were divided in four groups: PRGF, freeze-dried PRGF (PRGF lyo), and PRGF lyophilized mixed with 2,5% trehalose (PRGF lyo+2.5T) or 5% trehalose (PRGF lyof+5T). Chemical and biological characteristics were evaluated. Photorefractive keratectomy was performed on C57BL/6 mice which were divided in three treatment groups: control, PRGF, and PRGF lyo. Corneal wound healing and haze formation were evaluated macroscopically. Eyes were collected at 1, 2, 3, and 7 days after surgery, and were processed for histologic studies.

**Results:**

The pH values of PRGF samples increased significantly after the lyophilization process. Osmolarity levels increased significantly in PRGF samples mixed with trehalose in comparison with PRGF samples without protectants. The freeze drying process maintained growth factors levels as well as the biological properties of PRGF eye drops even without the use of lyoprotectants. PRGF lyo treatment significantly decreased the re-epithelialization time and haze formation in photorefractive keratectomy-treated corneas regarding PRGF and control groups. Furthermore, the PRGF lyo group significantly decreased the number of smooth muscle actin-positive cells in comparison with the control group at each time of the study and at days 2 and 3 in the PRGF group.

**Conclusions:**

The freeze drying process preserves the protein and growth factor content as well as the biological properties of PRGF eye drops, even without the use of protectants. Freeze-dried PRGF eye drops accelerate corneal tissue regeneration after photorefractive keratectomy in comparison with the control group.

**Translational Relevance:**

Our study shows the feasibility to preserve the biological capability of PRGF eye drops as freeze-dried formulation, avoiding the addition of protectants.

## Resumen (Foreign Language Abstract)

**Objetivo:**

Investigar si el colirio de plasma rico en factores de crecimiento (PRGF) mantiene su potencial biológico después de un proceso de liofilización. También se evaluó la adición de un lioprotector como la trehalosa.

**Métodos:**

Se recogió sangre de tres donantes sanos para obtener colirio mediante la tecnología PRGF. El colirio resultante se dividió en cuatro grupos: PRGF, PRGF liofilizado (PRGF lyo) y PRGF liofilizado mezclado con trehalosa al 2,5% (PRGF lyo + 2.5T) o trehalosa al 5% (PRGF lyof + 5T). Se evaluaron las características químicas y biológicas de cada uno de los colirios. Se realizó una queratectomía fotorrefractiva (PRK) en ratones C57BL/6 que se dividieron en tres grupos de tratamiento: Control, PRGF y PRGF lyo. La curación de la herida corneal y la formación de “haze” se evaluaron macroscópicamente. Los ojos se recolectaron en los días 1, 2, 3 y 7 después de la cirugía, y se procesaron para la realización de estudios histológicos.

**Resultados:**

Los valores de pH de las muestras de PRGF aumentaron significativamente después del proceso de liofilización. Los niveles de osmolaridad aumentaron significativamente en las muestras de PRGF mezcladas con trehalosa en comparación con las muestras de PRGF sin protectores. El proceso de liofilización mantuvo los niveles de factores de crecimiento, así como las propiedades biológicas de los colirios de PRGF, incluso sin el uso de lioprotectores. El tratamiento con PRGF lyo redujo significativamente el tiempo de reepitelización y la formación de “haze” en las córneas tratadas con PRK con respecto al grupo de control y PRGF. Además, el grupo PRGF lyo redujo significativamente el número de células SMA positivas en comparación con el grupo control en cada tiempo de estudio y en los días 2 y 3 con respecto al grupo de PRGF.

**Conclusiones:**

el proceso de liofilización conserva el contenido de proteínas y factores de crecimiento, así como las propiedades biológicas del colirio de PRGF, incluso sin el uso de protectores. El colirio de PRGF liofilizado acelera la regeneración del tejido corneal después de la PRK en comparación con el grupo de control.

**Relevancia Traslacional:**

nuestro estudio muestra la viabilidad de preservar la capacidad biológica del colirio de PRGF como formulación liofilizada evitando la adición de protectores.

## Introduction

A wide variety of diseases induce ocular epithelial and stroma injuries leading to several ocular surface disorders. The effective healing of these lesions is essential and necessary to recover the damaged ocular tissue functionality. The artificial tears are the most commonly used method for the treatment of ocular surface diseases. Nevertheless, artificial tears lack most of the biological features of natural tears, such as proteins, vitamins, pH, and osmolarity. In addition, they often contain preservatives, stabilizers, and other additives that can potentially induce toxic or allergic reactions.^1^

An interesting alternative to artificial tears is the use of blood derivatives. Eye drops obtained from plasma rich in growth factors (PRGF) represent a potential therapeutic method for the treatment of ocular surface pathologies. In addition to their lubricating properties, eye drops derived from PRGF technology contain a wide range of proteins that are involved in ocular regeneration, among which are several growth factors such as platelet derived growth factor, fibroblast growth factor, epidermal growth factor, and transforming growth factor-beta, among others.^2^^,^^3^

This biological method consists of the elaboration of a platelet-enriched plasma obtained from the patient's own blood to treat different pathologies from several medical fields.^4^^–^^7^ After activation, multiple bioactive proteins are released from the platelets, promoting several biological processes as cell recruitment, proliferation, migration, and cell differentiation.^8^^–^^10^

In recent years, PRGF eye drops have been widely used in the treatment of several ocular surface diseases, including dry eye, corneal ulcers, and persistent epithelial defects.^11^^–^^18^ The biochemical and biophysical properties of PRGF eye drops like pH, osmolarity, proteins, and growth factors content, which are similar to the artificial tears, are responsible for the successful results obtained in the treatment of these different ocular pathologies.^19^^,^^20^

Ocular surface disorders are usually chronic diseases that require medium- or long-term treatment. Therefore, the biological functionality and stability of these treatments need to be maintained for long periods of time to be used daily for months. In the case of blood-derived eye drops, several studies have shown their safety and stability over several months; however, their long-term storage and throughout the application period require the use and dependence of a cold chain (−20°C storage to keep it for a long period of time and +4°C or room temperature during use).^21^^,^^22^

In contrast, although autologous eye drops are commonly used for the treatment of ocular disorders, some patients are not suitable to be donors owing to systemic inflammatory diseases, age, and other types of disorders or comorbidities. The possibility of having available an allogeneic blood-derived product could be an interesting alternative to treat several ocular surface diseases in these types of patients.^23^

In both autologous and allogeneic products, eye drops lyophilization could be presented as an alternative to achieve a longer shelf-life for these products, avoiding a cold chain dependence. The freeze drying process often modifies protein structures because of the low temperature and the higher solute concentration owing to the freezing procedure.^24^ Some excipients such as trehalose are commonly added to the product to protect the proteins from stress during processing and storage. The effectiveness of trehalose is related to its capability to replace some water molecules preventing uncontrolled dehydration and promoting the protein stabilization.^25^

The main objective of the present study was to investigate whether PRGF eye drops preserves their biological potential after undergoing a lyophilization process. The effect of the administration of a lyoprotectant on the PRGF eye drops before the lyophilization process was also analyzed. In addition, the regenerative effects of these preparations on mice cornea after photorefractive keratectomy (PRK) surgery were evaluated.

## Methods

### Endoret Preparations

Blood from three healthy donors was collected after informed consent into 9-mL tubes with 3.8% (wt/v) sodium citrate. The study was performed following the principles of the Declaration of Helsinki. Samples were centrifuged at room temperature in an Endoret System centrifuge (BTI Biotechnology Institute, S.L., Vitoria, Spain). The whole plasma column was collected using Endoret ophthalmology kit (BTI Biotechnology Institute, S.L.) avoiding the layer containing leukocytes. Platelets and leukocytes counts were performed with a hematology analyzer (Micros 60, Horiba ABX, Montpelier, France). Whole platelet-rich plasma volume was activated with Endoret activator (BTI Biotechnology Institute, S.L.). The growth factor enriched supernatants obtained from each donor was aliquoted in glass vial for lyophilization and divided in four groups: (1) PRGF: PRGF supernatant (used as a control), (2) PRGF lyo: Pure PRGF supernatant frozen at −80°C, (3) PRGF lyo + 2.5T: PRGF supernatant was mixed with 2,5% trehalose as lyoprotectant and frozen at −80°C; and finally, (4) PRGF lyof + 5T: PRGF supernatant was mixed with 5% trehalose and then it was frozen at −80°C. All samples frozen at −80°C were introduced in the lyophilizer (LyoBeta, Telstar, Terrassa, Spain) to carry out the freeze-drying process. The primary drying phase was carried out at −50°C and 0.1 mBar for 24 hours. Finally, secondary drying phase was performed at +20°C and 0.1 mBar for 12 hours. Then, lyophilized samples were stored at + 4°C until use. Finally, the different freeze-dried PRGF eye drops samples were reconstituted with sterilized distilled water until getting the original volume before be used in the in vitro and in vivo assays.

### Percentage of Water Loss

Percentage of water loss was calculated by weighing the different PRGF eye drops samples before and after the freeze-drying process. Dry weight was subtracted from each sample after their incubation for 24 hours at 60°C. Then, the water loss percent was calculated using the following equation:

$$\begin{array}{c} \mathrm{Water}\;\mathrm{loss}\;\%=\left(W_{0}-W_{l}\right)/W_{0}\times 100, \end{array}$$

where W_0_ and W_l_ is the weight of PRGF eye drops before and after lyophilization, respectively.

### Characterization of PRGF Eye Drops

The concentration of several growth factors involved in the ocular surface tissue regeneration, such as epidermal growth factor, platelet-derived growth factor, transforming growth factor-beta 1, vascular endothelial growth factor and insulin-like growth factor type I were analyzed to evaluate the lyophilization process effect on the PRGF eye drops. These growth factors were analyzed using the commercial enzyme-linked immunosorbent assay kits purchased from R&D Systems (Minneapolis, MN). The pH was analyzed with a pH meter (Hach Lange Spain; Barcelona, Spain), and the osmolarity was measured with the Advanced Model 3320 Micro-Osmometer (Tecil, Barcelona, Spain). All of these analyses were performed on the different PRGF samples obtained from the three healthy donors.

### Cells

The biological activity of eye drops samples obtained in the present study were evaluated in two primary cell types from the ocular surface, corneal keratocytes (called HK) and conjunctival fibroblasts (termed HConF) (ScienCell Research Laboratories, San Diego, CA). Both cell types were cultured according to manufacturer's instructions. Briefly, cells were cultured until confluence in fibroblast medium supplemented with fibroblast growth supplement (Complete FM; ScienCell Research Laboratories) and then were detached with animal origin-free trypsin like enzyme (TrypLE Select, Gibco-Invitrogen, Grand Island, NY). Cell viability was assessed by trypan blue dye exclusion.

### Proliferation Assay

The ocular surface cells were seeded in a 96-well dark bottom plate at a density of 10,000 cells/cm^2^ in serum-free medium supplemented with 20% (v/v) of the different PRGF samples obtained from the three donors. The study period was 72 hours. The cell density was analyzed using the CYQUANT cell proliferation assay (Invitrogen, Carlsbad, CA). Briefly, the culture medium was removed, and the wells were washed carefully with phosphate-buffered saline. Subsequently, the plate was frozen at −80°C to produce a better cell lysis efficiency in the CYQUANT assay. After thawing the plate at room temperature, the samples were incubated with RNase A (1.35 Ku/mL) diluted in cell lysis buffer for 1 hour at room temperature. Then the 2x CyQUANT GR dye/cell lysis solution was added to each well of the plate, mixed gently and incubated for 5 minutes at room temperature and protected from light. The fluorescence of the sample was measured using a fluorescence microplate reader (Twinkle LB 970, Berthold Technologies, Bad Wildbad, Germany). A DNA standard curve ranging from 7.8 to 1000.0 ng/mL was included in all fluorescence quantifications. Calibration curves ranging from 2500 to 90,000 cells/cm^2^ were established in each assay as an index of cell number (CyQUANT assay; Invitrogen).

### Migration Assay

To quantify the migratory potential of the ocular surface cells after treatment with the different PRGF eye drops samples obtained in the present study, the cells were seeded at high density inside the culture inserts (Ibidi, GmbH, Martinsried, Germany) placed in a 24-well plate and were grown with fibroblast growth supplement (Complete FM; ScienCell Research Laboratories) until confluence. Then the inserts were carefully removed, and two separated cell monolayers were obtained leaving a cell-free gap of approximately 500 µm thickness. The cells were washed with phosphate-buffered saline and incubated in quintuplicate with the different eye drops for 24 hours. After this period, the different culture media were removed, and the cells were incubated with Hoechst 33342 at a dilution of 1:500 in phosphate-buffered saline for 10 minutes. To quantify the number of migrated cells, phase contrast images were taken from the central part of the gap before treatment and phase contrast and fluorescence after 24 hours of treatment using a digital camera coupled to an inverted microscope (Leica DFC300 FX and Leica DM IRB, Leica Microsystems, Barcelona, Spain). Image J software (National Institutes of Health, Bethesda, MD) was used to measure the gap area taken in each image and the number of cells migrated after 24 hours.

### PRK Mouse Model

A total of 120 male C57BL/6 mice were used in this study. All procedures were performed in accordance with the tenets established in the directive of the European Parliament and Council of the European Communities (2010/63/ UE), of the ARVO Statement, and of the Spanish legislation (RD 1201/2005 and Law 32/2007) for the Use of Animals.

Surgical injury was performed using an Allegretto WaveLight excimer laser (PR-020407, Wavelight GmbH, Alcon, Erlangen, Germany) as previously described.^26^^–^^30^ Right eyes were subjected to PRK surgery with a 2.0-mm ablation zone on the central cornea and a depth of 45 µm. The procedure included the simultaneous ablation of the epithelium (using a defined epithelial thickness profile of 25 µm centrally) and 20 µm of the anterior stroma (about 20% of the total stromal thickness) in a single step. Before the surgical procedures, mice were deeply anesthetized by intraperitoneal injection of a mixture of ketamine hydrochloride (80 mg/kg; Imalgene 1000, Merial Laboratorios S.A., Barcelona, Spain) and xylazine hydrochloride (5 mg/kg; Rompun, Bayer Hispania S.L., Barcelona, Spain) followed by topical application of 0.5% tetracaine chlorhydrate and 1 mg of oxybuprocaine (Colircusí Anestésico Doble, Alcon S.A., Barcelona, Spain).

Animals were divided in three groups: in the control group, mice were treated with saline solution (Fresenius Kabi, Barcelona, Spain); in the PRGF group, they were treated with whole fraction of PRGF-Endoret eye drops; and in the PRGF lyo group, they were treated with lyophilized whole fraction of PRGF-Endoret and reconstituted with sterile water at the time of use. In the in vivo studies, each PRGF formulation (PRGF and PRGF lyo) from the three donors was pooled before treating the different eyes to normalize the treatments. The treatment was administrated topically six times per day along the first 3 days, and four times per day until the end of the study. The evolution of the ocular surface damage in each group was analyzed at four time points: 1, 2, 3, and 7 days of treatment.

### Macroscopic Analysis

Corneal wound healing was examined under a Leica S6D stereoscopic microscope equipped with an EC3 digital camera (Leica Microsystems) using a fixed magnification of 12.5×. Observations were made immediately after injury and at 1, 2, 3, and 7 days after PRK surgery, and calibrated images were obtained from each eye. The fluorescein staining test was used to estimate the area (in square millimeters) of the corneal epithelial defect using FIJI image analysis software (ImageJ 1.48d; National Institutes of Health). Briefly, the region of fluorescein-impregnated cornea was manually lined with the freehand selection tool of FIJI on calibrated images. The software automatically calculated the area inside the selection. Previous to fluorescein staining, the level of opacity in the cornea (haze grade) was assessed in a double-blinded fashion by two researchers observing under the stereomicroscope, according to the Fantes scale,^29^ scoring from 0 (clear) to 4 (severely dense opacity).

### Tissue Collection and Histopathologic Analysis

Mice were euthanized under general anesthesia with an overdose of sodium pentobarbital (Dolethal, Vétoquinol, Lure, France) injected intraperitoneally at each time point of the study (1, 2, 3, and 7 days). For histologic analysis, eyes were enucleated and immersed in buffered 4% paraformaldehyde and 0.2% picric acid for 1 hour at room temperature, cryoprotected in 30% sucrose, embedded in OCT compound (Optimum Cutting Temperature; Tissue-Tek, Sakura, Tokyo, Japan), and snap frozen in liquid nitrogen. Transversal 5-µm-thick sections were obtained with a Microm HM550 cryostat (Microm International GmbH, Walldorf, Germany) through the central region of the cornea for immunofluorescence analysis. Immunofluorescence assays were performed as described previously using antibodies to alpha-smooth muscle actin (α-SMA) (myofibroblast transformation; 1:200; Abcam) and Ki67 (proliferation marker; 1:500; Abcam).^26^^,^^27^^,^^30^^,^^31^ Samples were incubated overnight with the corresponding antibody and revealed with an Alexa Fluor 594 anti-rabbit secondary antibody (1:500; Molecular Probes, Eugene, OR). To detect DNA fragmentation associated with apoptosis, terminal deoxyribonucleotidyl transferase-mediated dUTP-fluorescein nick-end labeling (TUNEL) was performed on frozen sections according to the manufacturer's instructions (Promega Corp., Madison, WI). Nuclei were counterstained with 4’,6-diamidino-2-phenylindole (DAPI; 2 µg/mL; Molecular Probes). Sections were examined under a Leica DM 6000 fluorescence microscope equipped with a digital image capture system (Leica Microsystems).

### Quantification of Immunofluorescence and TUNEL Assay

Three sections (5 µm thick and spaced with 50 µm between them) obtained from the central region of five corneas for each time point and experimental group were used to quantify Ki-67–positive proliferative cells, alpha-smooth muscle actin–positive myofibroblasts, and TUNEL apoptotic cells. Two independent observers counted every positive cell with clearly identifiable nuclei stained in blue with DAPI on five non-overlapping corneal regions of 224.14 × 167.38 µm in size. Each microscope field comprised a column of central corneal tissue extending from the anterior epithelium to the posterior stromal surface.

### Statistical Analysis

A general linear model repeated-measures analysis of variance was used to analyze the differences among the PRGF eye drops obtained in the present study for the different variables studied. The significance of the post hoc comparisons was calculated with the Bonferroni correction. In the case of an in vivo assay, the nonparametric Kruskal-Wallis test with a subsequent Mann-Whitney analysis test for multiple comparisons between groups were used. A difference at a level of a *P* of less than 0.05 was considered to be statistically significant. Statistical analyses were performed using SPSS software (version 15.0; SPSS Inc., Chicago, IL).

## Results

The mean platelet enrichment of the Endoret preparations was 2.14-fold over the baseline concentration of platelets in whole blood. The PRGF platelet concentration obtained from the three donors ranged from 284 to 434 × 10^3^ platelets/µL. None of the preparations contained detectable levels of leucocytes.

### Freeze-Drying of PRGF Eye Drops

**Figure 1.**
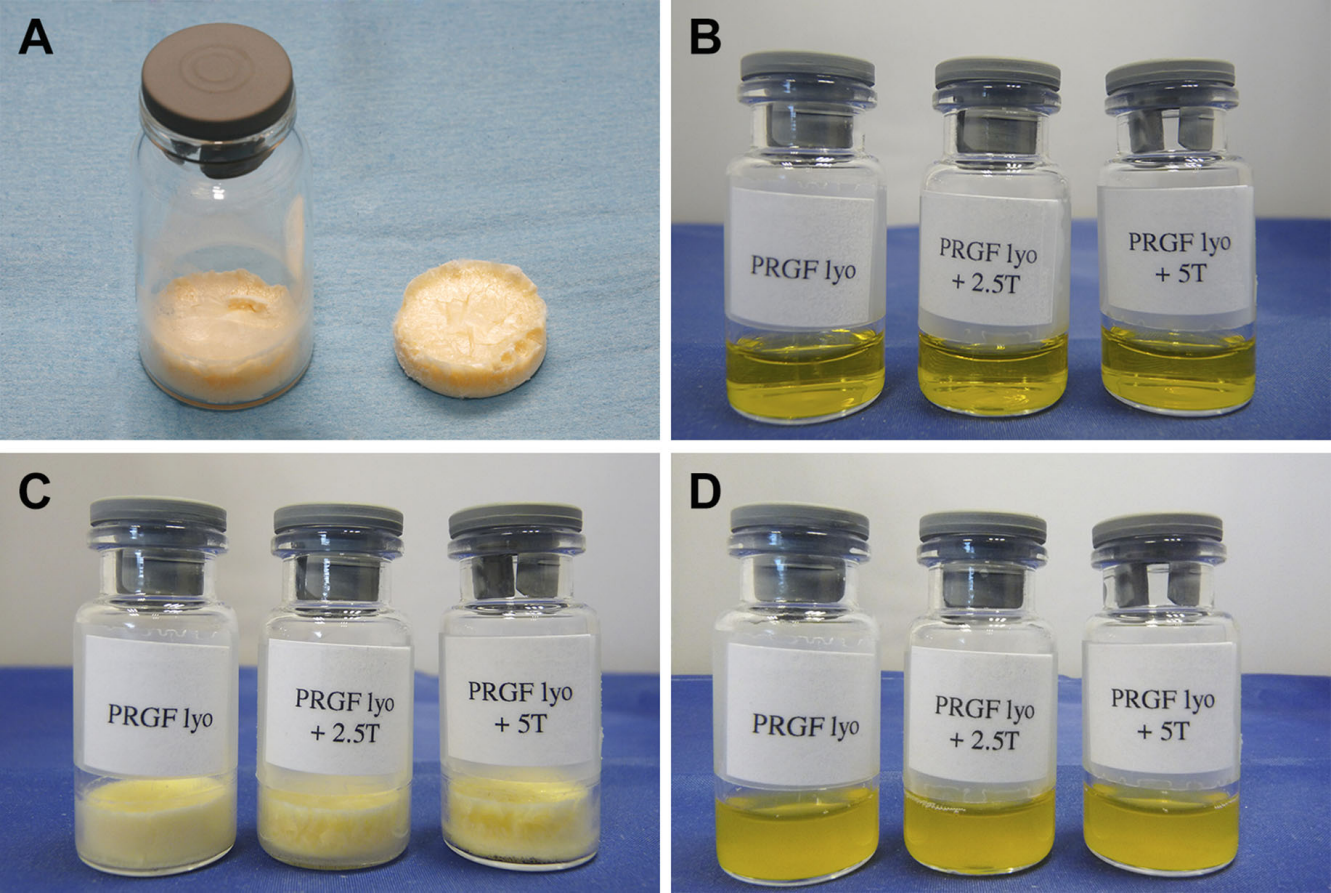
Representative images of the PRGF eye drops appearance along the freeze-dried procedure. (A) A kind of yellowish homogeneous wafer was obtained after PRGF lyophilization. Different PRGF eye drops samples analyzed along the study (PRGF lyo, PRGF lyo+2.5T, and PRGF lyo+5T) before their lyophilization (B), after the freeze-dried process (C), and after their reconstitution with equal bulk of distilled water to the original volume (D).

After PRGF eye drops lyophilization a kind of homogeneous soft wafer (Fig. 1A) was obtained that it can be easily broken getting a yellowish powder. The wafers obtained from the three groups of lyophilized PRGF eye drops (Fig. 1B: PRGF lyo, PRGF lyo+2.5T, and PRGF lyo+5T) had similar morphologic and color features (Fig. 1C). The different lyophilized PRGF eye drops samples were rehydrated in an equal volume of distilled water to return them to their original concentration. The reconstitution of each sample was almost instantaneous obtaining a homogeneous and cloudy solution in all samples used in the present study (Fig. 1D).

### Percentage of Water Loss

**Figure 2.**
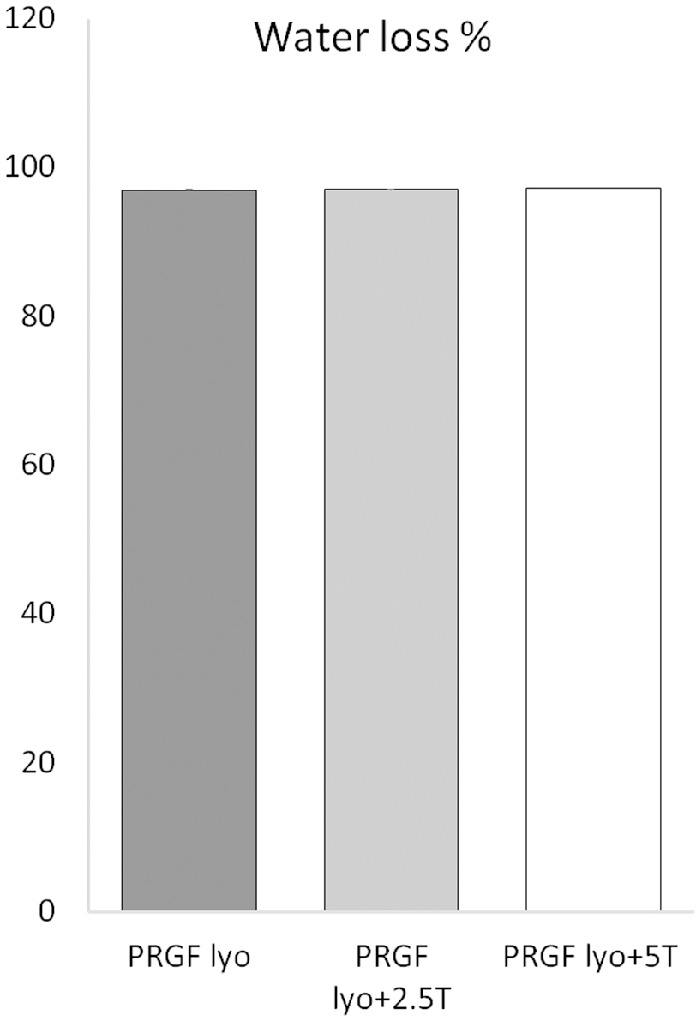
Percentage of water loss after the lyophilization process of the different PRGF eye drops samples obtained in the study.

No significant differences (*P* > 0.05) were observed in the percentage of water loss among the different freeze-dried PRGF eye drops samples obtained along the study. As it is shown in Figure 2, the percentage of water loss in freeze-dried PRGF eye drops (PRGF lyo) reached a 97.19%, similar to the water loss obtained in those PRGF eye drops mixed with trehalose at 2.5% or 5% achieving also a 97.18% and 97.22% of water loss, respectively.

### Characterization of PRGF Eye Drops

The concentration of several growth factors involved in ocular surface regeneration were analyzed in the PRGF eye drops stored at −20°C (PRGF), freeze-dried PRGF without lyoprotectants (PRGF lyo) and mixed with 2.5% trehalose (PRGF lyo+2.5T) or 5% (PRGF lyo+5T). The levels of the different growth factors analyzed are presented in Table 1. The results shown that the concentration of the different growth factors analyzed were kept constant after lyophilization of the PRGF eye drops with or without lyoprotectant with respect to the control group (Table 1). The measurement of pH in the different PRGF eye drops samples showed that lyophilization process increased the pH levels becoming statistically significant (*P* < 0.05) in comparison to fresh PRGF samples (PRGF, PRGF+2.5T and PRGF+5T) (Table 2). No significant differences were observed among the fresh PRGF eye drops samples mixed or not with trehalose. However, significant differences (*P* < 0.05) were observed among the different freeze-dried PRGF eye drops samples, reaching the highest pH values in the freeze-dried PRGF eye drops samples (PRGF lyo) (pH of 8.54), followed by PRGF+2.5T samples (pH of 8.32) and PRGF lyo+5T samples (pH of 8.20). Osmolarity values increased significantly (*P* < 0.05) in PRGF samples mixed with trehalose in comparison to PRGF without lyoprotectans (PRGF and PRGF lyo) (Table 2). This osmolarity level increase was correlated with the trehalose percentage applied to PRGF eye drops. Osmolarity values increased in fresh samples from 319 in PRGF to 394 and 488 in PRGF+2.5T and PRGF+5T, respectively, whereas in the freeze-dried samples the levels increased from- 301 in PRGF lyo group to 376 in PRGF lyo+2.5T and 440 in PRGF lyo+5T. However, the results showed that the lyophilization process maintained the osmolarity values between the fresh PRGF samples and their corresponding freeze-dried samples.

**Table 1.** Concentrations of Several Growth Factors in the Different PRGF Eye Drops

	PDGF-AB (pg/mL)	TGF-β1 (ng/mL)	VEGF (pg/mL)	EGF(pg/mL)	IGF(ng/mL)
PRGF (*n* = 3)	17,300 ± 3204	23,963 ± 1879	196 ± 92	556 ± 18	90 ± 3
PRGF lyo (*n* = 3)	15,485 ± 5413	20,950 ± 841	174 ± 135	493 ± 86	84 ± 4
PRGF lyo+2.5T (*n* = 3)	15,683 ± 4902	22,138 ± 4038	164 ± 81	509 ± 2	83 ± 1
PRGF lyo+5T (*n* = 3)	14,133 ± 3220	20,463 ± 4946	171 ± 53	555 ± 59	80 ± 10

EGF, epidermal growth factor; IGF, insulin-like growth factor; PDGF, platelet-derived growth factor; TGB-β1, transforming growth factor-beta1; VEGF, vascular endothelial growth factor.

Data represent mean ± standard deviation. No significant differences (*P* > 0.05) were observed in the levels of the growth factors analyzed among the different samples of PRGF eye drops.

**Table 2.** pH and Osmolarity Values (mean ± SD) Measured in Each PRGF Eye Drop Samples

	PRGF	PRGF+2.5T	PRGF+5T	PRGF lyo	PRGF lyo+2.5T	PRGF lyo+5T
pH	7.53 ± 0.09^a,b,c^	7.59 ± 0.08^a,b,c^	7.58 ± 0.09^a,b,c^	8.54 ± 0.13*^,^^†^^,‡,b,c^	8.32 ± 0.18*^,^^†^^,‡,a,c^	8.20 ± 0.18*^,^^†^^,‡,a,b^
Osmolarity (mOsm/Kg)	319 ± 7^†^^,‡,b,c^	394 ± 7*^,‡,a^	488 ± 19*^,^^†^^,a,b,c^	301 ± 6^†^^,‡,b,c^	376 ± 6*^,‡,a,c^	440 ± 18*^,‡,a,b^

Data represent mean ± standard deviation. *Statistically significant regarding PRGF (*P* < 0.05). ^†^Statistically significant respecting PRGF+2.5T (*P* < 0.05). ^‡^Statistically significant regarding PRGF+5T (*P* < 0.05). ^a^Statistically significant with respect to PRGF lyo (*P* < 0.05). ^b^Significant differences with respect to PRGF lyo+2.5T (*P* < 0.05). ^c^Statistically significant with respect to PRGF lyo+5T (*P* < 0.05).

### Biological Activity of Freeze-Dried PRGF Eye Drops

**Figure 3.**
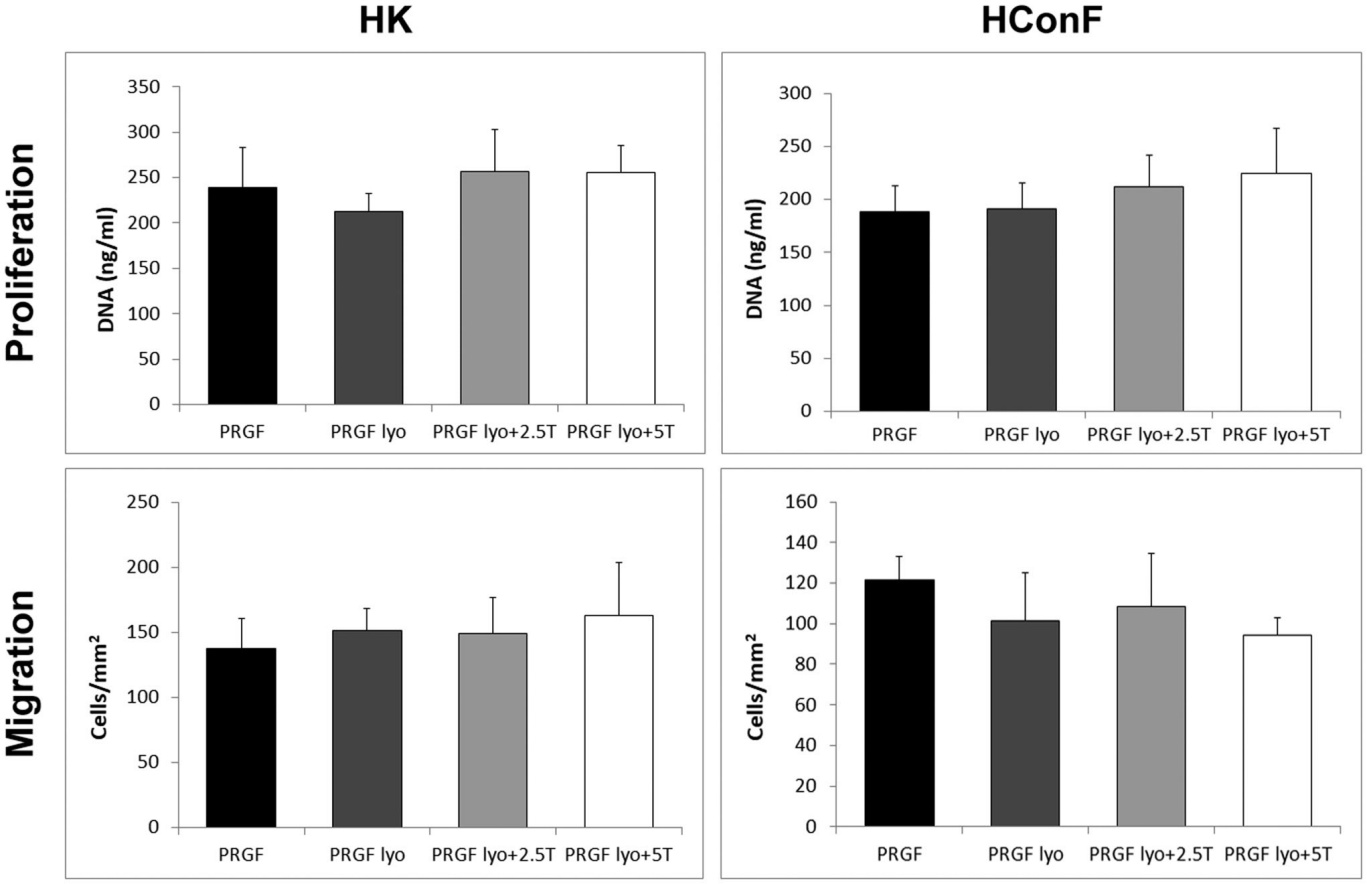
Biological activity of corneal keratocytes (HK) and conjunctival fibroblasts (HConF) after culture with PRGF eye drops (PRGF; *n* = 3) or freeze-dried PRGF without lyoprotectans (PRGF lyo; *n* = 3) or mixed with 2.5% (*n* = 3) or 5% (*n* = 3) trehalose (PRGF lyo+2.5T and PRGF lyo+5T, respectively). No significant differences were observed in the proliferation index, nor in the migration activity of the HK and HConF cells after being cultured with the different study samples.

The biological activity was measured as proliferative and migratory potential of freeze-dried PRGF eye drops samples in HK and HConF cells. Both types of cells showed no significant differences in proliferation and migration levels after treatment with the different PRGF eye drops samples obtained along the study (Fig. 3).

### Macroscopic Analysis

**Figure 4.**
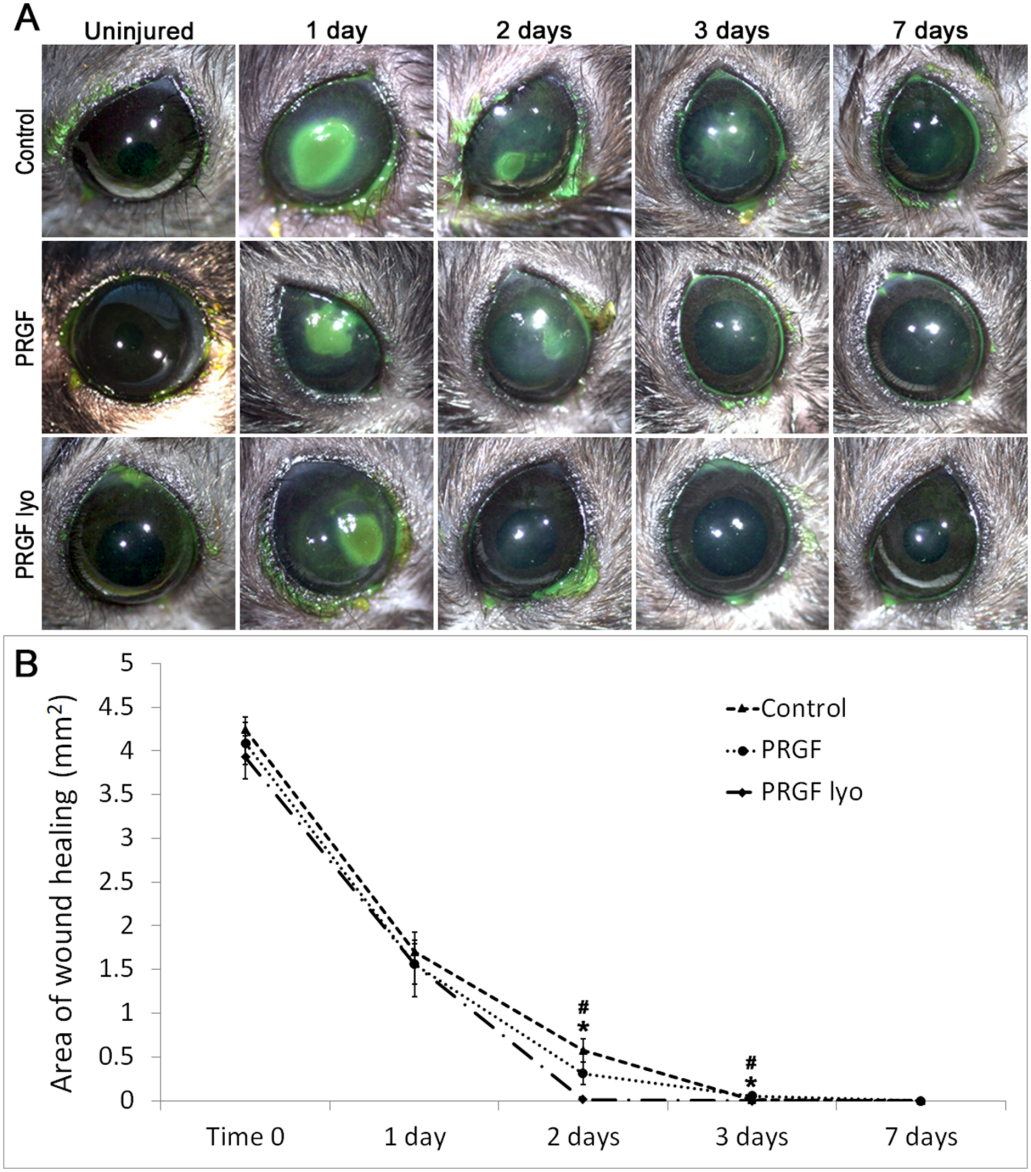
Macroscopic analysis of corneal wound healing stained with fluorescein after PRK surgery. (A) Representative images of corneal wound healing at different time points of the study of the different treatment groups. At the end of the study (day 7) slightly corneal fluorescence staining were observed in some mice of the control group. (B) Significant differences in the area of corneal wound healing were observed in mice treated with PRGF lyo at 2 and 3 days after surgery in comparison with the control and PRGF groups. At day 3, all of the mice corneas treated with PRGF lyo were completely closured, whereas in the control group at least 20% of the corneas remained unclosed at the end of the study (day 7). Results have been expressed as a mean area of wound healing ± standard error of the mean (*n* = 10 per treatment group). *Statistically significant differences between the PRGF lyo and control groups (*P* < 0.05). **^#^**Statistically significant differences between PRGF lyo and PRGF group (*P* < 0.05).

No adverse reactions were found on mice ocular surface tissues after treatment with human reconstituted freeze-dried PRGF eye drops. Partial re-epithelization was observed in all mice eyes at the first time point (day 1) showing no differences among the treatment groups (Fig. 4A). On the second day, the fluorescein positive corneal lesion area was significantly decreased in the PRGF lyo–treated eyes compared with PRGF and control eyes (*P* < 0.01) (Fig. 4B). Furthermore, 55% of the eyes treated with PRGF lyo presented a total re-epithelization, whereas only 20% of control eyes showed successfully closed epithelium at day 2. However, none of the mouse corneas treated with PRGF were completely closed at day 2 of treatment. All eyes in PRGF lyo group were completely re-epithelized at day 3, whereas 40% of the control group and a 45% of the PRGF group corneas remained damaged. Moreover, 20% of the eyes in the group treated with saline showed small focus of fluorescein stained defects at the end of the study (day 7) (Fig. 4A). In the case of the PRGF group, all corneas were completely re-epithelized at day 7.

**Figure 5.**
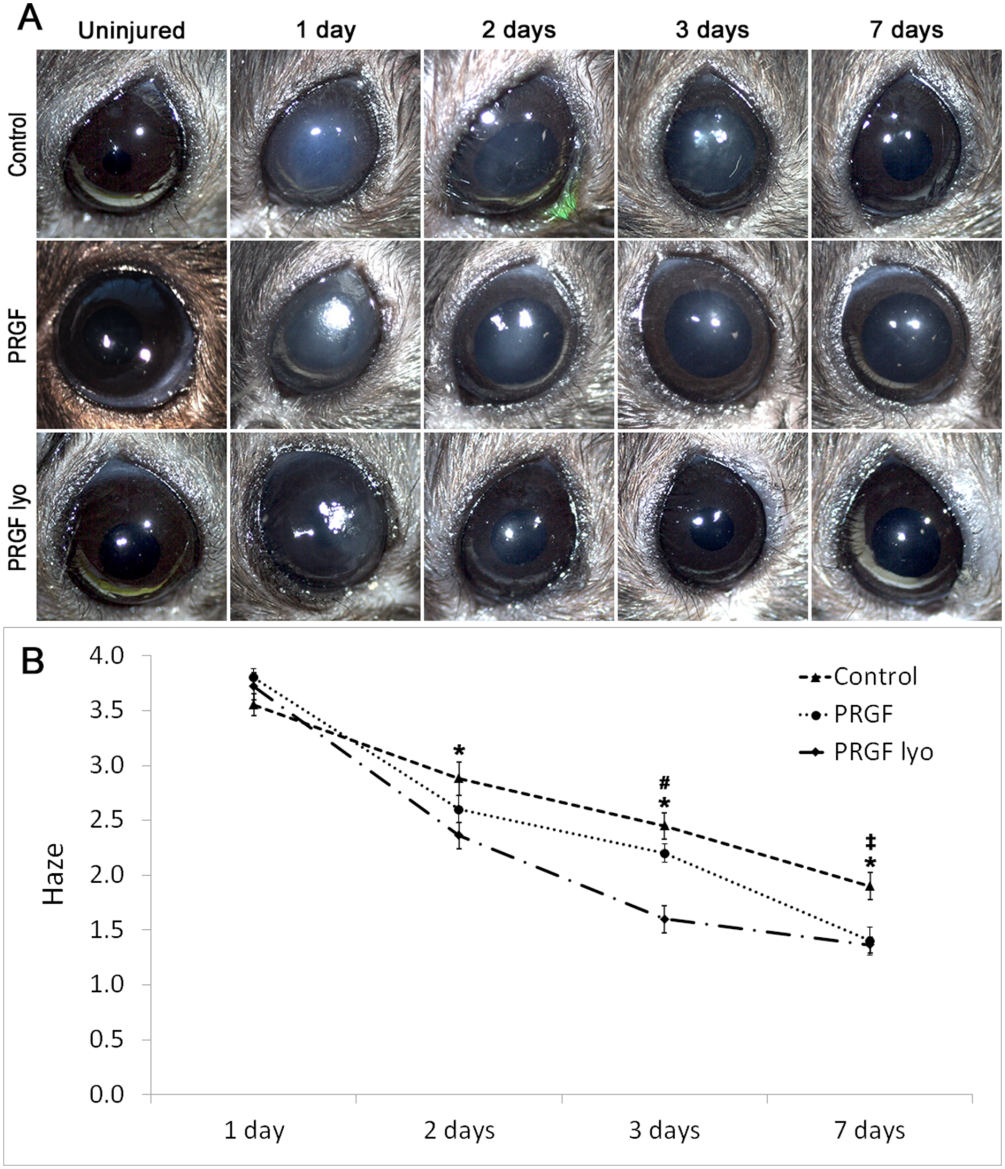
Macroscopic analysis of haze development after PRK surgery. (A) Representative images of mice corneas from the different treatment groups at different times of the study. PRGF lyo and PRGF treatments decreased corneal haze formation showing transparent corneas at days 3 and 7 after PRK surgery, respectively. However, corneal haze was observed along the study in mice corneas of the control group. (B) Haze development was significantly decreased in corneas treated with PRGF lyo eye drops regarding those treated with control from day 2 to the end time of the study. In addition, corneas treated with PRGF lyo showed a significant reduction in the corneal haze formation in comparison to PRGF group at day 3 of treatment. In contrast, haze formation was significantly reduced after PRGF treatment regarding control group at day 7 of the study. Results have been expressed as a mean haze grade ± standard error of the mean (*n* = 10 per treatment group). *Statistically significant differences between the PRGF lyo and control groups (*P* < 0.05). **^#^**Statistically significant differences between PRGF lyo and PRGF group (*P* < 0.05). **^‡^**Statistically significant differences between PRGF and control group (*P* < 0.05).

At the same time, PRK-treated corneas were analyzed in the different treatment groups to evaluate the haze development at each time point (Fig. 5A). The mouse corneas treated with PRGF lyo showed lower grade of stromal haze than control group from day 2 to the end of the study. Thus, corneas treated with PRGF lyo were significantly (*P* < 0.05) more transparent than in saline group at days 2, 3, and 7 after PRK (Fig. 5B). Moreover, haze formation was significantly reduced after PRGF lyo treatment in the PRGF group at day 3 of the study. In contrast, PRGF treatment decreased significantly haze formation in comparison to the control group at day 7 of treatment (Fig. 5B).

### Histologic Analysis

#### Proliferation

**Figure 6.**
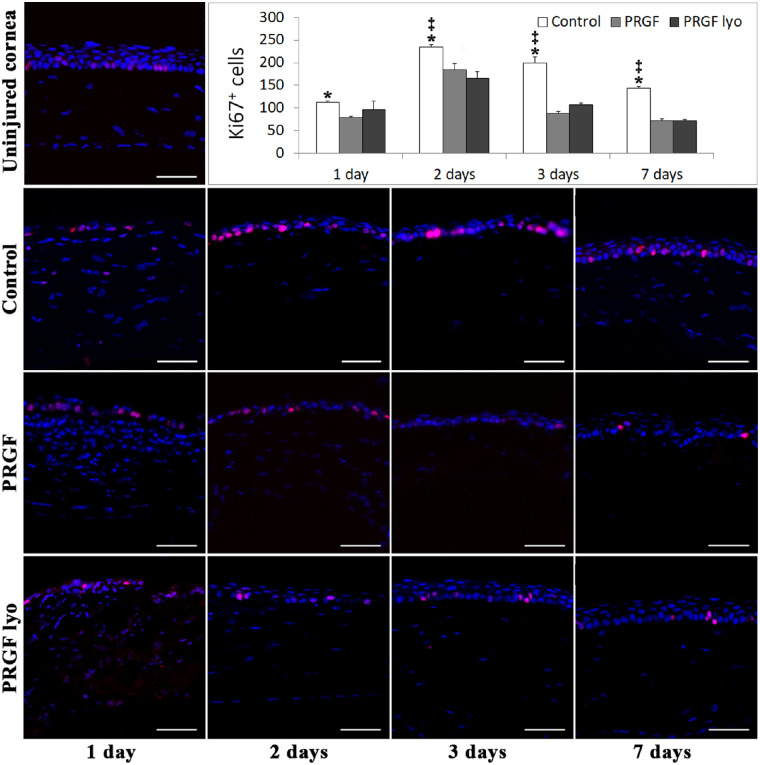
Histologic analysis of Ki-67–positive cells after PRK surgery. Representative images of the central area of the cornea of uninjured cornea and the different treatment groups at each time point of the study showing Ki-67–positive cells stained in red and cell nuclei in blue. Control treatment increased significantly the number of Ki-67–positive cells (mean ± standard error of the mean) in mice corneal epithelium at each time of the study regarding PRGF group and at 2, 3, and 7 days from surgery in comparison to the PRGF lyo treatment. *n* = 10 per treatment group and per study time. *Statistically significant differences in comparison to PRGF lyo group (*P* < 0.05). **^‡^**Statistically significant differences with respect to PRGF group (*P* < 0.05). Scale bar: 50 µm.

Ki-67–positive cells increased drastically in all mice corneal epithelium near to limbal area at day 1 after PRK surgery, showing a wave of Ki-67–positive cells from the periphery to the center of the cornea. At this point, the center of the cornea was not covered by epithelium, but some Ki-67–positive cells seemed to organize in rows at the surface of the injured area (Fig. 6). At day 1 of treatment, statistically significant differences (*P* < 0.05) were only found between PRGF-treated and control corneas in the number of epithelial Ki-67–positive cells, whereas no differences were found between the corneas treated with PRGF lyo and control group at this time in the study. The greatest epithelial proliferation rate was observed at day 2 after injury, decreasing progressively the number of Ki-67–positive cells until the end of the study. PRGF lyo and PRGF corneas exhibited a significantly (*P* < 0.05) lower number of proliferative cells at 2, 3, and 7 days after surgery compared with control corneas. Seven days after injury, the number of Ki-67–positive cells in the mice corneas treated with PRGF (71.4 ± 4.86 cells) and with PRGF lyo (71.62 ± 2.49 cells) was near the normal number of proliferative cells in an uninjured cornea (58.66 ± 4.41 cells), whereas the number of proliferative cells in saline group was three times higher (144.50 ± 3.83 cells).

#### Apoptosis

**Figure 7.**
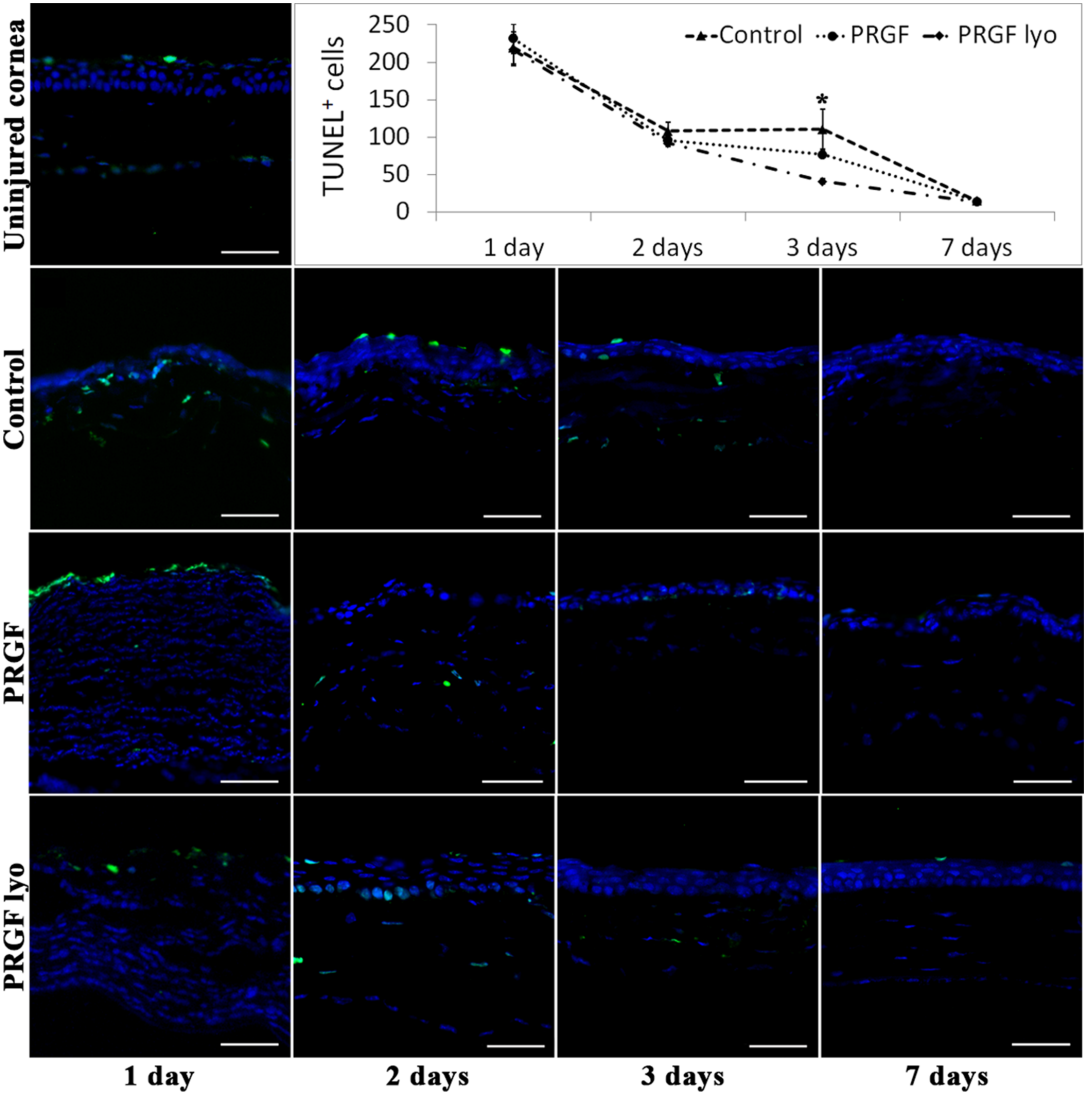
Histologic analysis of apoptotic cells using TUNEL immunofluorescence technique after PRK surgery. Representative images of the central area of the cornea of uninjured cornea and the different treatment groups at each time point of the study. A high number of TUNEL-positive cells stained in green were detected at day 1 after surgery in mice corneal stroma, cell nuclei are stained in blue. The number of TUNEL+ cells (mean ± standard error of the mean) were progressively decreasing until reaching similar levels of untreated corneas at day 7 from surgery. *n* = 10 per treatment group and per study time. Significant differences (*,*P* < 0.05) were observed between control and PRGF lyo treatment groups at day 3 after PRK surgery. Scale bar: 50 µm.

TUNEL-positive cells were detected in high number at day 1 after surgery in mice corneal stroma (Fig. 7). The number of apoptotic cells decreased progressively until basal levels at day 7. At this moment, TUNEL-positive cells were only detected at the superficial epithelial layer of flat cells. No statistical differences were detected among the treatment groups in the number of apoptotic cells excepting for 3-day time point, where PRGF lyo–treated eyes showed a significant reduced number of TUNEL-positive cells compared with control treated eyes (*P* < 0.05).

#### Immunofluorescence Detection of Myofibroblasts

**Figure 8.**
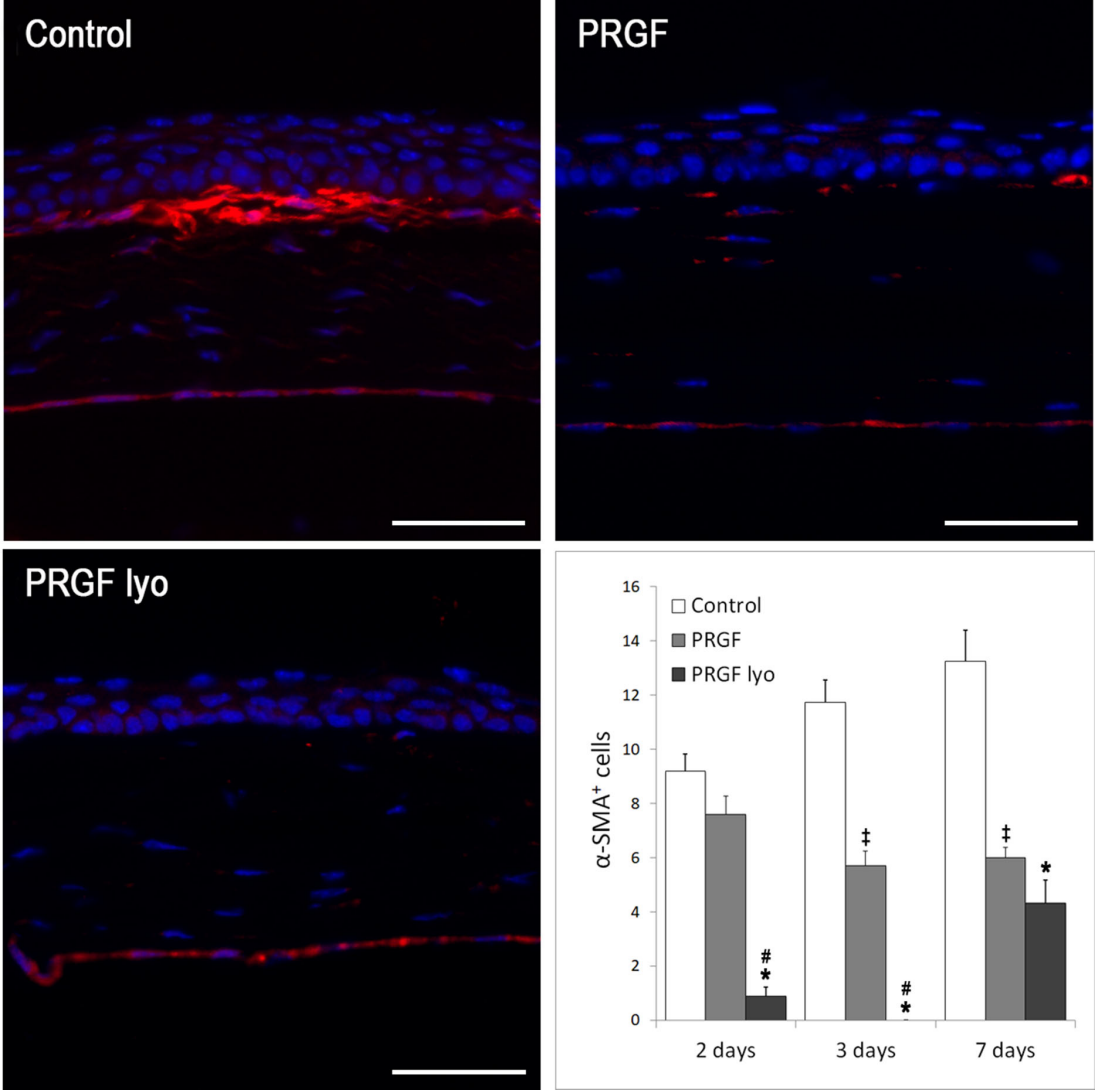
Representative images of α-SMA immunofluorescence of the different treatment groups at 7 days after PRK surgery. A higher number of SMA-positive cells were observed in control mice corneas regarding PRGF lyo and PRGF. At this time of treatment, SMA-positive cells were just detected underneath corneal epithelium. PRGF lyo decreased significantly (*P* < 0.05) the number of SMA positive cells in comparison to control group at 2, 3 and 7 days after PRK surgery and regarding PRGF treatment at day 2 and 3. SMA-positive cells were significantly decreased after treatment with PRGF in comparison with the controls at days 3 and 7 after PRK surgery. Results are expressed as the mean number of SMA-positive cells ± standard error of the mean. *n* = 10 per treatment group and per study time. *Statistically significant differences between PRGF lyo and control group (*P* < 0.05). **^#^**Statistically significant differences between PRGF lyo and PRGF group (*P* < 0.05). **^‡^**Statistically significant differences between PRGF and control group (*P* < 0.05). Scale bar: 50 µm.

The first α-SMA positive cells identified as myofibroblasts were observed at day 2 after injury, being the amount of α-SMA–positive cells significantly higher (*P* < 0.001) in the control group (9.20 ± 0.63 cells) and in the PRGF group (7.60 ± 0.67) compared with scarce cells observed in PRGF lyo group (0.88 ± 0.35 cells) (Fig. 8). Only 44% of the eyes in the group treated with PRGF lyo presented α-SMA immunoreactivity in the stroma while 100% of the control and PRGF groups showed α-SMA–positive cells.

At day 3 the number of myofibroblasts in the control group increased (11.73 ± 0.83 cells). However, the number of α-SMA–positive cells was decreased in PRGF and PRGF lyo-treated corneas, showing a lack of α-SMA staining cells in the mice corneas treated with PRGF lyo at this time point. Significant differences were observed in the number of SMA positive cells in the corneas treated with PRGF and PRGF lyo regarding the control group at day 3. In addition, PRGF lyo decreased significantly the number of cells stained with SMA in comparison with the PRGF group at this time of the study.

Seven days after surgery, there was a significant decrease (*P* < 0.05) in the quantity of α-SMA–positive myofibroblasts in PRGF lyo (4.33 ± 0.83 cells) and PRGF (6.00 ± 0.38) treatment groups compared with control (13.25 ± 1.16 cells; *P* < 0.001). Myofibroblasts were located at the uppermost layers of the anterior stroma, forming a thick layer in contact with the basal epithelial cells in the control group (Fig. 8). Most of the myofibroblasts were observed in zones where the inferior epithelial border was not regular or presented a non-mature aspect. Corneas from PRGF and PRGF lyo groups showed a restored multilayered epithelium with an organized and linear basal cell layer and presented a low amount of myofibroblasts.

## Discussion

The tear film is composed of a wide content in electrolytes, mucins, proteins, and peptides that confer it particular characteristics such as lubrication, biological activity and antimicrobial effect.^32^ In the last years, the development of a product similar to the natural tear has reached a great interest to be used in the treatment of the ocular surface diseases. The use of blood derivative products is not new and their beneficial effects in the ophthalmology field is attributed to their properties and protein content similar to the natural tears. It contains a wide range of functional proteins such as growth factors and vitamins, that regulate proliferation, migration, and differentiation processes of the ocular surface cells.^33^

In recent decades, several studies have demonstrated the effectiveness and safety of the autologous blood-derived eye drops as an alternative treatment for several ocular surface disorders.^12^^,^^18^^,^^34^^,^^35^ However, autologous blood donation is not suitable for some patients owing to systemic inflammatory diseases, age, and other types of disorders or comorbidities. An allogeneic blood-derived product could be an alternative for these patients to treat several ocular surface diseases.^23^ Furthermore, in some countries like the UK, Germany, and New Zealand the production of blood-derived eye drops is regulated by different regulatory agencies, and its manufacturing is centralized in hospitals or blood banks.^36^ This situation causes some concerns to the patients as the need to travel from the specialist's office to the nearest hospital and to wait during hours in the hospital until the drops are ready.^36^ In fact, some blood derivatives manufacturing take 2 to 3 days and also in some countries like Germany competent authorities might require to quarantine eyedrop preparations for bacterial contamination testing, which could take up to 1 to 2 weeks.^37^ Therefore, allogeneic blood derived eye drops could be an alternative for supplying hospitals to solve the problems derived from the use and manufacture of autologous products. A high quantity of “off-the-shelf” allogeneic blood derived eye drops can be produced, improving the logistics of the treatment.^38^ In recent years, the New Zealand Blood Service Clinical Advisory Group and the regulatory authority in New Zealand (Medsafe) has approved the use of allogeneic blood derived eye drops when the use of autologous eye drops was not feasible or clinically possible.^39^

The freeze-drying process could be an alternative to increase the shelf-life of this blood derived product and to facilitate the accessibility in an off-the-shelf manner.

In the present work, we have observed that water content after PRGF eye drops lyophilization was less than 5% considering this percentage as an adequate in a freeze-dried process.^40^ Then, the different lyophilized PRGF eye drops samples were reconstituted with a bulk of distilled water equal to their original volume. The chemical analyses of the different samples showed that pH values increased significantly after lyophilization until reaching their highest values to 8.54 in the case of PRGF lyo. However, preclinical and clinical studies have demonstrated that eye drops formulations should have a pH between 6.6 and 9.0 to avoid irritation.^41^ Our results show that pH in all of the PRGF samples analyzed remained under 9.0 value indicating that they are perfectly tolerated by the ocular tissues.

The normal tear has an osmolarity levels ranged between 300 and 310 mOsm/kg.^42^ Various clinical studies have demonstrated that osmolarity levels higher than 425 mOsm/kg induce an inflammatory response on ocular surface cells triggering a discomfort feeling in patients with dry eyes.^43^ Our results show that pure PRGF eye drops samples maintain the osmolarity values in similar levels to those of the normal tears even after the lyophilization process. However, PRGF eye drops samples mixed with trehalose could exert an adverse effect on ocular surface tissues in patients with dry eye owing the higher osmolarity levels achieved after the addition of protectants.^43^^,^^44^

In recent years, several studies have shown that lyophilization of different blood-derived products mixed with lyoprotectants like trehalose maintain their biological properties.^45^ However, in this study, we have demonstrated that PRGF eye drops can be lyophilized without the use of lyoprotectants maintaining all of their chemical and biological properties.

Similar results were observed in the in vivo model where PRGF lyo demonstrated faster wound healing than PRGF and control groups. The cause of the higher re-epithelialization capability of PRGF lyo in contrast with the PRGF could be due to the higher migratory activity of PRGF lyo after its storage for a few months at 4°C or at room temperature.^46^ In this sense, some proteins or growth factors involved in the control/inhibition of cell migration could be partially or totally denaturalized during freeze-drying process or during the storage period reducing the re-epithelialization time of mice corneas treated with PRGF lyo. However, further studies will be needed to evaluate the proteins and growth factors involved at this study point. However, the re-epithelization capacity of the treatment groups observed in in vivo models was not correlated with the number of proliferative cells (ki-67–positive) at each time of treatment. The faster re-epithelization of PRGF lyo is probably due to the higher migratory capability of PRGF eye drops in comparison to the control treatment as it was observed in a previous study.^27^ Furthermore, this increase in the epithelial cell proliferation of the control group could be related with the irregular epithelia observed at the end of the study in contrast to the mice corneal epithelium treated with PRGF and PRGF lyo, where it was closely similar to the native corneal epithelium.

In addition, the higher corneal haze observed in the control and PRGF groups in comparison with the PRGF lyo–treated mice from the second to seventh day of treatment were correlated with the detection of SMA+ cells in the different treatment groups. PRGF lyo eye drops decreased significantly (*P* < 0.05) the number of SMA-positive cells on stromal corneas regarding the control group at 2, 3, and 7 days of treatment, whereas this significant decrease was observed between the PRGF lyo group and the PRGF group at days 2 and 3 of treatment. The presence of myofibroblast cells (SMA-positive cells) is highly related with the development of corneal haze, which is correlated with the results observed by biomicroscopic analysis in the different groups at each time of the study.

A TUNEL assay showed no differences among groups of treatment, except at day 3, where a significant decrease in the number of apoptotic cells were observed in the corneas treated with PRGF lyo in comparison with the control group. This difference was due to the no changes observed in the number of apoptotic cells between days 2 and 3 after surgery in the control group in contrast with the PRGF group, where the number of apoptotic cells were progressively decreasing along the study time.

Although further investigation is needed, the present results suggest that the lyophilization process could increase the shelf-life of the PRGF eye drops and could improve the shipping and storage procedure in comparison with the actual storage temperature restrictions.^21^^,^^46^

## Conclusions

The results obtained in the present study show that freeze-dried PRGF eye drops maintain their protein and growth factors content as well as their biological properties, even without the use of lyoprotectants. The therapeutic effects of lyophilized PRGF eye drops may be comparable to those of fresh PRGF eye drops in the treatment of ocular surface disorders. Freeze-dried PRGF eye drops could be an alternative as an off-the-shelf product to be used as an allogeneic eye drops for the treatment of several ocular surface diseases.
